# Reindeer Warble Fly–associated Human Myiasis, Scandinavia

**DOI:** 10.3201/eid1905.130145

**Published:** 2013-05

**Authors:** Boris Kan, Kjetil Åsbakk, Kristian Fossen, Arne Nilssen, Rosario Panadero, Domenico Otranto

**Affiliations:** Karolinska University Hospital, Stockholm, Sweden (B. Kan); Norwegian School of Veterinary Science, Tromsø, Norway (K. Åsbakk);; University Hospital of Northern Norway, Tromsø, Norway (K. Fossen);; Tromsø University Museum, Tromsø (A. Nilssen);; Universidad de Santiago de Compostela, Lugo, Spain (R. Panadero);; Università degli Studi di Bari, Valenzano, Italy (D. Otranto)

**Keywords:** Hypoderma tarandi, reindeer warble fly, human, Scandinavia, parasites, myiasis

**To the Editor:** We report migratory myiasis that occurred during 1991–2012 caused by the reindeer warble fly, *Hypoderma tarandi* (Technical Appendix Figures 1, 2), in 7 tourists to reindeer habitats of northern Scandinavia. We also report 2 additional women (patients 8 and 9), independent of each other, who were asymptomatic but sought medical care in August 2012 after finding 30–60 parasite eggs in scalp hair 3 days after hiking in Kebnekaise and Jämtland Mountains (northern Sweden), respectively.

Patients 1–7 (Table) had enlarged regional lymph nodes and migratory dermal swelling of the head and upper face. Rounded cutaneous swelling of 2–5 cm occurred 1 at a time, persisted for 1–3 days, and reappeared after 2–34 days.

**Table:** Myiasiss caused by warble reindeer fly (*Hypoderma tarandi*), Scandinavia, 1991–2012*

Characteristic	Case-patient no.						
	1	2	3	4†	5	6	7‡
Age, y/sex	8/M	10/M	10/M	10/M	6.5/F	56/F	28/M
Date of symptom onset	2008 Sep 15	2009 Aug 15	2010 Sep 12	2010 Sep 3	2010 Dec 3	2011 Oct 10	1991 Aug
Signs and symptoms	Enlarged occipital and retroauricular lymph nodes	Eggs in scalp hair; enlarged occipital and cervical lymph nodes	Forehead swelling	Enlarged occipital and cervical lymph nodes	Forehead and eyebrow swelling	Occipital swelling	Occipital swelling
Travel							
Dates	2008 late Jul–early Aug	2009 Jul 20–Aug 7	2010 Jul 7–16	2010 Aug 14–19		2011 Jul	1991 Jul
Destination	Norway, extreme northeast: patient resides in Troms County where reindeer are occasionally seen	Sweden: Lapland	Sweden: Kiruna, Riksgränsen, Abisko (several short hiking tours) Norway: Bodö	Sweden: family undertook a 5-d hiking tour in Lunndörrsfjällen, a mountain area in Jämtland County		Norway: Lapland	Sweden: short trips to Arvidsjaur (Lapland) and Jämtland Mountains
Observed reindeer	Yes	Yes§	No	Yes; at close range		Yes	Yes
Migratory swellings, no.	Temple, 1	Head, 1; forehead, 5	Forehead, 4; eyelid, 1; behind ear, 1	Forehead and eyelid, 5	Forehead and eyelid, 2	Head, temple, eyelid, >5	Head, >5
Fever	Yes	No	No	Yes	No	No	No
Eosinophilia (highest value)¶	Yes (1,0)	Yes (0,6)	Yes (0,8)	Yes (3,8)	No	No	Unknown
Other signs and symptoms	Uveitis, failure to gain weight	Localized exanthema	Itching of scalp, enlarged retroauricular lymph nodes, headache, uveitis	Fever, headache, nausea	No	Enlarged nuchal lymph nodes	Uveitis, glaucoma, retinal hemorrhage
Diagnostic delay, d#	74	25	14	0	0	1	>60
Diagnosis	Positive serology, morphologic identification of larva	Positive serology, identification of eggs	Positive serology, molecular identification of larva	Positive serology	Positive serology	Negative serology**	Negative. serology in 2011 and 2012, morphologic identification of larva in 1991
Drugs received							
Ivermectin	No	5 doses	5 doses††	5 doses	3 doses	2 doses	No
Antihistamines	No	Yes	Yes	Yes	Yes	Yes	Unknown
Oral steroids	Yes	No	No	Yes	Yes	Yes	Steroids, antimicrobial drugs given after surgery
Outcome	After eye surgery glaucoma; visual acuity 0,9	Good	Eye surgery; visual loss, right eye	Good	Good	Good	Eye surgery; visual loss, right eye

*Patients 8 and 9 are not included in the table because myiasis did not develop in them. †Patients 4 and 5 are siblings. ‡Patient 7 was discovered by the father of patients 4 and 5 among his acquaintances, suggesting the possibility of additional unreported cases in the population. §In Jukkasjärvi (Sweden), the child had visited an enclosure where the reindeer were agitated because of swarms of flies. ¶Referent 0–0.5 × 10^9^/L. #Interval between date of first visit for myiasis-associated symptoms and date when treatment began. **The diagnosis could not be confirmed, but her clinical picture and response to treatment were similar to those of other patients. ††Ivermectin was given first after eye surgery.

In mid-January 2009, 4 months after initial symptoms, patient 1 felt a sudden pain in his left eye; 10 days later, an ophthalmologist discovered an intraocular larva (Technical Appendix Figure 3). Patient 3 had a swelling on his forehead, which reappeared 2× before his right eyelid swelled; the day after the eyelid swelling disappeared, vision decreased in his right eye. Patients 1, 3, and 7 underwent eye surgery; 1 living larva was extracted from each patient. Patients 3 and 7 lost vision in the affected eye.

For 5 patients, ivermectin was administered orally (≈200–350 µg/kg body weight), on 3–5 occasions in relation to the swellings. Patients 8 and 9 also each received 1 dose of ivermectin; they remained asymptomatic. Patient 3 received the first dose on day 5 after the living larva was extracted because of a new swelling. Swelling recurred on 3 occasions 2 weeks–1.5 months after surgery. In patient 7, swelling reappeared on several occasion 10–30 days after eye surgery, indicating that retrieval of 1 larva does not exclude concomitant occult infestations. This probably was also the case for patient 2, who had a swelling on his upper forehead when pain developed at the root of his nose, where a new swelling appeared 4 days later.

The 3 larvae removed from patients 1, 3, and 7 were identified as *H. tarandi*, 2 by morphology and 1 by molecular-specific amplification and sequencing (*1*). Antibodies against hypodermin C, an enzyme released by the larva during migration in host tissues, were detected in 5 of the symptomatic patients (*2*,*3*). 

*H. tarandi* eggs take 4–7 days to hatch, depending on the temperature of the hair layer (*4*); thus, patients 8 and 9 were treated soon after oviposition and were seronegative. Newly hatched *H. tarandi* larvae can easily dry, so their chance of survival is higher when they are close to scalp skin. Eggs from patient 2 were initially misidentified as head lice eggs but were eventually identified as *H. tarandi* by T.G. Jaenson (Uppsala). Published photographs of the *H. tarandi* eggs alongside the eggs of head lice (5) helped identify *H. tarandi* eggs in patients 8 and 9. According to those patients, *H. tarandi* eggs could not be removed from the hair with a lice comb. The *H. tarandi* fly is well adapted to sub-Arctic climate; nearly all reindeer were found to be infested in some districts of northern Finland and Norway (*6*). Reindeer habitats attract tourists, mostly during summer. *H. tarandi* is mainly active on warm summer days; warm weather perhaps does not encourage persons to cover their heads, which may predispose for oviposition. Also, persons moving around probably attract more flies than do those staying still, and strong wind, rain, and temperatures <10°C–12°C are thought to inhibit the warble fly’s flight activity and oviposition (*7*).

Awareness of human infestation by *H. tarandi* warble flies increased in Sweden and Norway after news media in Sweden described patient 2 (*5*; http://www.lakartidningen.se/engine.php?articleId=14643). This publication helped in the recognition of symptoms and in shortening diagnostic delay in patients 3–6, 8, and 9. Of the 3 cases for which diagnosis was not delayed, patients 4 and 5 were children of a physician who read our publication and recognized the symptoms; patient 6, herself a physician, also read the article (5). Increased awareness, rather than increased incidence, explains the emergence of new cases. Nine of 12 cases of proven *H.* *tarandi* myiasis found in the literature occurred in persons who had ophthalmomyiasis interna (*3*,*8*,*9*); migratory dermal swellings, the clinical signature of hypodermosis, have been reported only in 1 case (*10*). Such swellings occurred in all the patients reported here, suggesting that clinicians overlooked this finding, possibly because of the overtaking severity of eye complications and the reporting of most previous cases by ophthalmologists (*3*,*8*,*9*). Persons who seek care for migratory dermal swellings during August–December should be asked about recent travel to reindeer habitats.

For 3 patients with ophthalmomyiasis reported here, ophthalmologists initially had difficulty establishing a diagnosis, raising the possibility that some cases of “idiopathic” uveitis from *H. tarandi*–endemic areas may be caused by *H. tarandi*. Ophthalmomyiasis should be considered in cases of unilateral uveitis, lens subluxation, and suspicion of intraocular foreign body (*3*,*8*,*9*). Eosinophilia might be absent and should not be used to guide treatment.

Technical Appendix Female *Hypoderma tarandi* warble fly in flight, eggs of warble fly on a reindeer hair, and intraocular larvae crawling on retinal surface of patient 1.
