# Supplementary material for: Reindeer Warble Fly–associated Human Myiasis, Scandinavia
Source: Emerg Infect Dis. 2013 May;19(5):830–2. doi: 10.3201/eid1905.130145 (PMC3647520; doi:10.3201/eid1905.130145)
Supplement: Technical Appendix — Female Hypoderma tarandi warble fly in flight, eggs of warble fly on a reindeer hair, and intraocular larvae crawling on retinal surface of patient 1. [file 13-0145-Techapp-s1.pdf]

Article DOI: http://dx.doi.org/10.3201/eid1905.130145

Reindeer Warble Fly–associated Human Myiasis, Scandinavia

Technical Appendix

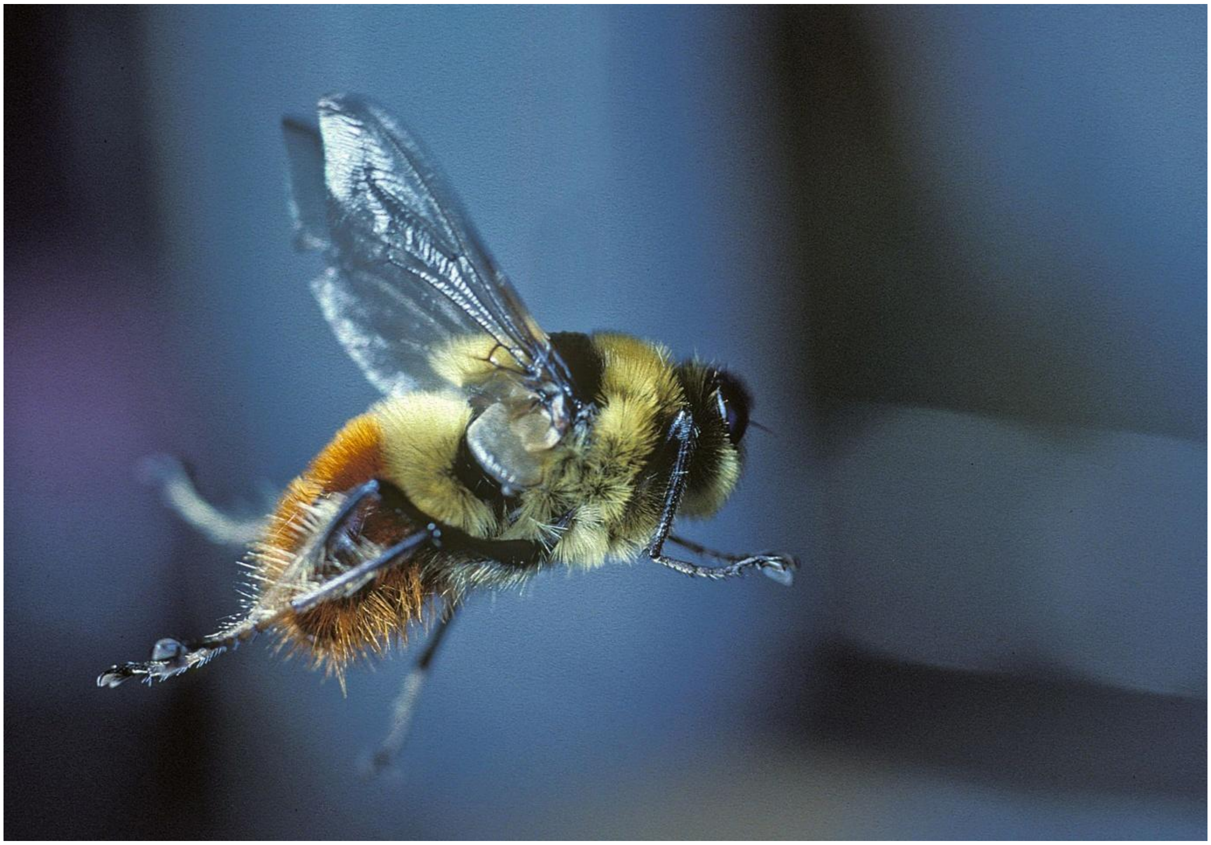

Technical Appendix Figure 1. Female *Hypoderma tarandi* reindeer warble fly in flight. The parasite can fly for many hours and may cover 600–900 km during its lifetime. This flight capacity has evolved to find reindeers. They do not feed as adults. Photo by A. Nilssen.

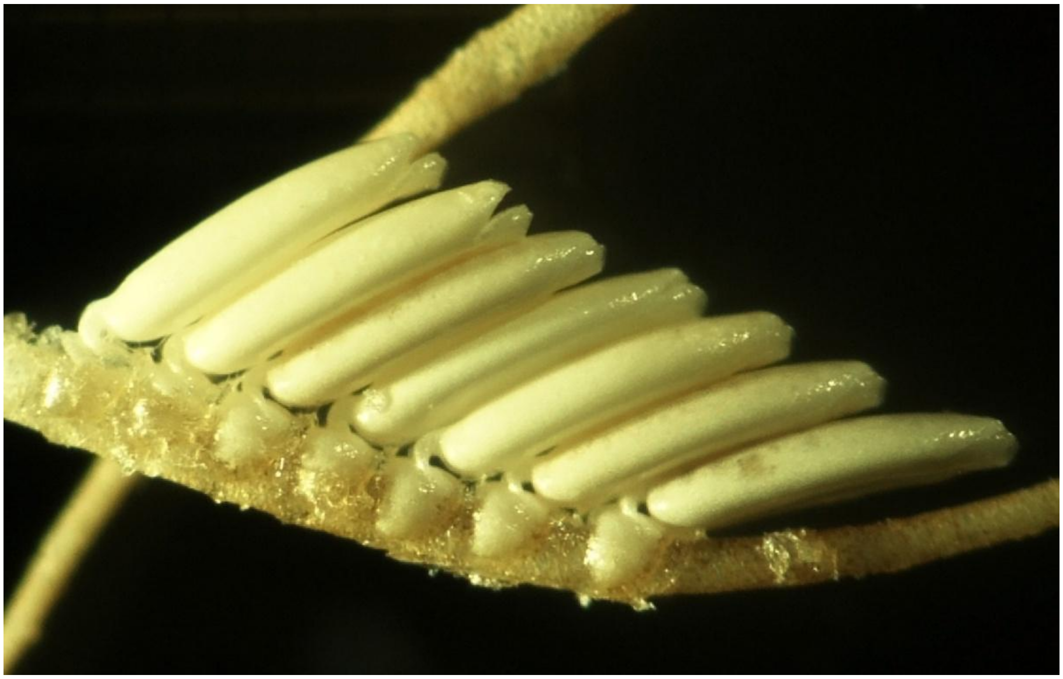

Technical Appendix Figure 2. Eggs of *Hypoderma tarandi* stick to a reindeer hair with an attachment organ and a glue-like liquid. Upon hatching, the larvae borrow through the skin near the root of the hair shaft. Photo by K. Åsbakk.

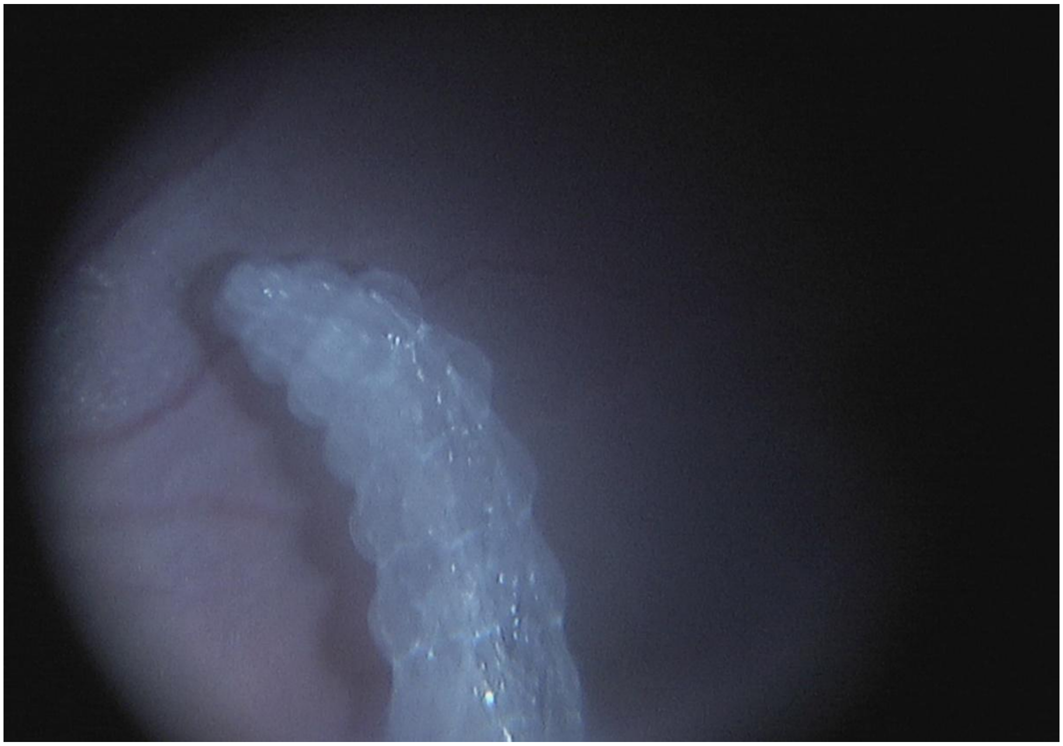

Technical Appendix Figure 3. Intraocular larvae crawling on retinal surface of patient 1. Photo taken intraoperatively by K. Fossen.
